# Expanded HIV pre-exposure prophylaxis (PrEP) implementation in communities in New South Wales, Australia (EPIC-NSW): design of an open label, single arm implementation trial

**DOI:** 10.1186/s12889-017-5018-9

**Published:** 2018-02-02

**Authors:** Iryna B. Zablotska, Christine Selvey, Rebecca Guy, Karen Price, Jo Holden, Heather-Marie Schmidt, Anna McNulty, David Smith, Fengyi Jin, Janaki Amin, David A. Cooper, Andrew E. Grulich, on behalf of the EPIC-NSW study group, Andrew E. Grulich, David A. Cooper, Iryna B. Zablotska, Rebecca Guy, Janaki Amin, Fengyi Jin, Christine Selvey, Jo Holden, Heather-Marie Schmidt, Bill Whittaker, Karen Price, Nic Parkhill, Kerry Chant, Craig Cooper, Levinia Crooks, Debbie Allen, David Baker, Mark Bloch, Rohan Bopage, Katherine Brown, Andrew Carr, Christopher Carmody, Kym Collins, Robert Finlayson, Rosalind Foster, Eva Jackson, David Lewis, Josephine Lusk, Anna McNulty, Catherine O’Connor, Nathan Ryder, David Smith, Emanuel Vlahakis, Phillip Read, Barbara Yeung, Gesalit Levitt, Erin Ogilvie, Stefanie Vaccher, Muhammad Hammoud, Lucy Watchirs-Smith, Nasir Wabe

**Affiliations:** 1 The Kirby Institute, University of New South Wales, Sydney, 2052 Australia; 2 NSW Ministry of Health, Sydney, NSW Australia; 3 AIDS Council of New South Wales (ACON), Sydney, NSW Australia; 4 Sydney Sexual Health Centre, Sydney, NSW Australia; 5 Mid North Coast Local Health District (Area HIV/Sexual Health Services), Lismore Health Service, Lismore, NSW Australia; 6 Macquarie University, Sydney, NSW Australia

**Keywords:** HIV risk, Pre-exposure prophylaxis, PrEP eligibility, Implementation research, Gay, homosexual or other men who have sex with men, Antiretroviral medication, HIV incidence, adherence

## Abstract

**Background:**

The New South Wales (NSW) HIV Strategy 2016–2020 aims for the virtual elimination of HIV transmission in NSW, Australia, by 2020. Despite high and increasing levels of HIV testing and treatment since 2012, the annual number of HIV diagnoses in NSW has remained generally unchanged. Pre-exposure prophylaxis (PrEP) is highly effective in preventing HIV infection among gay and bisexual men (GBM) when taken appropriately. However, there have been no population-level studies that evaluate the impact of rapid PrEP scale-up in high-risk GBM. Expanded PrEP Implementation in Communities in NSW (EPIC-NSW) is a population-level evaluation of the rapid, targeted roll-out of PrEP to high-risk individuals.

**Methods:**

EPIC-NSW, is an open-label, single-arm, multi-centre prospective observational study of PrEP implementation and impact. Over 20 public and private clinics across urban and regional areas in NSW have participated in the rapid roll-out of PrEP, supported by strong community mobilization and PrEP promotion. The study began on 1 March 2016, aiming to enroll at least 3700 HIV negative people at high risk of HIV. This estimate took into consideration criteria for PrEP prescription in people at high risk for acquiring HIV as defined in the NSW PrEP guidelines. Study participants receive once daily co-formulated tenofovir disoproxil fumarate and emtricitabine (TDF/FTC) and are followed for up to 24 months. Follow-up includes: testing for HIV at 1 month, HIV and other sexually transmissible infections three-monthly, HCV annually and monitoring of renal function six-monthly. Optional online behavioural surveys are conducted quarterly. The co-primary endpoints are (i) HIV diagnoses and incidence in the cohort and (ii) HIV diagnoses in NSW.

**Discussion:**

EPIC-NSW is a population-based PrEP implementation trial which targets the entire estimated population of GBM at high risk for HIV infection in NSW. It will provide a unique opportunity to evaluate the population impact of PrEP on a concentrated HIV epidemic.

**Trial registration:**

https://clinicaltrials.gov/ (identifying number NCT02870790; registration date 14 August 2016), pre-results stage.

**Electronic supplementary material:**

The online version of this article (10.1186/s12889-017-5018-9) contains supplementary material, which is available to authorized users.

## Background

In New South Wales (NSW), Australia, research findings in HIV treatment as prevention [1] were followed in 2012 by a state-wide HIV strategy that had an over-arching aim of working towards the virtual elimination of HIV by 2020 [2]. In response to widespread implementation of policy actions directed towards HIV treatment as prevention [3], HIV testing rates and ART coverage among people diagnosed with HIV have increased considerably. By 2015, an estimated 93% of people with HIV in NSW were diagnosed; 89.3% of those diagnosed were on treatment, of which 91.3% had suppressed viral load over the preceding 12 months [4–6]. However, by the end of 2015, new HIV diagnoses in NSW had not declined substantially, and more than 80% of diagnoses continued to be in gay and bisexual men (GBM) [7]. Undiagnosed, and thus untreated, HIV infections among GBM [8], combined with increasing levels of condomless anal intercourse with casual partners [9], continued to drive the HIV epidemic despite very substantial increases in HIV testing and treatment. In order to achieve a significant reduction in new HIV diagnoses, additional strategies were required. In this context, the NSW HIV Strategy 2016–2020 proposed the population-level roll-out of HIV pre-exposure prophylaxis (PrEP), in combination with strengthening condom use, testing, and treatment, in order to move forward towards the ambitious goal of virtually eliminating HIV transmission by 2020 [10].

PrEP, in the form of combined tenofovir disoproxil fumarate 300 mg (TDF) and emtricitabine 200 mg (FTC), is effective for primary HIV prevention [11–13]. TDF/FTC has a favorable safety profile [14] and effectively penetrates the anorectal mucosa [15–17], which makes it an ideal prophylactic agent for GBM. PrEP efficacy is closely related to adherence [14], and adherence has been higher in people who are well-informed about HIV and PrEP [12, 13, 18]. TDF/FTC is now recommended as PrEP for people at high risk of HIV acquisition in national or regional guidelines in the US, Europe and Australia [19–21], as well as by the WHO guidelines globally [22].

At the time when EPIC-NSW began recruitment in March 2016, TDF/FTC as PrEP was not approved by the Australian Therapeutic Goods Administration (TGA) [23]. The first guidance for PrEP prescribing, which became available in NSW in October 2014 (formally published in 2016 [24]), defined the eligibility for PrEP in a PrEP demonstration project PRELUDE [25]. Funded by the NSW Government, PRELUDE provided access to PrEP to about 300 individuals, predominately GBM from November 2014–November 2016 [25]. However, PrEP demand rapidly surpassed the PRELUDE project’s capacity. Ongoing PrEP research in NSW [25–27], PrEP guidelines [24] and the new NSW HIV Strategy 2016–2020 [10] provided the framework for a population-level implementation project “Expanded PrEP Implementation in Communities in NSW”(EPIC-NSW).

While PrEP implementation among GBM has been in various stages of roll-out in other countries (e.g., in the US and France), EPIC-NSW is novel in being a hybrid research and implementation project targeting PrEP to all people at high risk of HIV infection in NSW, and measuring the public health benefit of this program with respect to reduction in HIV transmissions in the state.

This study protocol describes the population-level introduction of PrEP in NSW through a public health intervention, which is widely supported across the spectrum of researchers, clinicians, community organisations, and government policymakers.

## Methods / design

### Aim

EPIC-NSW aims to evaluate the population-level impact of the rapid roll-out of PrEP in individuals at high risk for HIV and to measure the impact on HIV incidence among GBM in NSW.

### Study design

EPIC-NSW is a prospective observational study of PrEP roll-out, implementation and impact. It is an open-label, single-arm, multi-centre trial, undertaken by a multidisciplinary team led by the Kirby Institute, UNSW Sydney. The target population is predominantly GBM at high risk of HIV acquisition through sexual contact. However, other high risk individuals who are eligible for PrEP under the NSW PrEP guidelines can also enrol in the study (see Table 1).

**Table 1:**
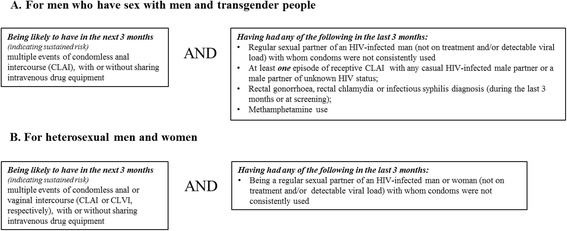
Risk criteria for participation in the EPIC-NSW trial

Note: According to the EPIC-NSW protocol, only people classified at high risk for HIV acquisition based on the NSW guidance on PrEP can be enrolled into the study

All participants are advised to take daily TDF/FTC and attend study visits as per the schedule presented in Table 2. Follow-up includes testing for HIV at month one, for sexually transmitted infections (STIs) and HIV every 3 months, and for hepatitis C annually. Renal function tests are conducted at baseline, at 3months after enrolment, and every 6 months thereafter. Participants are also invited to join the optional study components: (i) self-administered online surveys of behaviour and medication adherence (at baseline and quarterly thereafter) and (ii) data linkage with the Australian Government and state agency health and disease-related registries approved by the NSW Population and Health Services Research Ethics Committee. The planned enrolment is a minimum of 3700 participants. Enrolment started on 1 March 2016. The project anticipates a total of 7400 person-years (PY) of follow-up on PrEP (up to 2 years of follow-up per person). To evaluate the uptake and impact of PrEP on the HIV epidemic in NSW, EPIC-NSW utilizes extraction of routinely collected data from electronic records maintained in clinical patient management systems across all sites using data extraction methods set up by the Australian Collaboration for Coordinated Enhanced Sentinel Surveillance (ACCESS study) [28].

**Table 2:** EPIC-NSW study flowchart

Follow-up schedule	Screening/enrolment/baseline	Follow-up 1	Subsequent Follow-ups
Timeline	Week −2 or 0^a, b^	Month 1	Month 3 and every 3 months thereafter^c^
Informed consent	X		
Review eligibility	X	X	X
Medical history^d^	X		
Renal function (creatinine clearance)	X		X^e^
Serious adverse events, pregnancy (if applicable)		X	X
Hepatitis C testing	X		X^f^
STI testing ^g^	X		X
HIV testing^g^	X	X	X
Dispense study medication	X	X	X
Survey of adherence and behaviour	X		X^h^

^a^Results of all tests conducted in the preceding 2 weeks are accepted at enrolment. Only the HIV test should be conducted not earlier than 7 days within starting PrEP

^b^Visits may be combined if HIV test result available

^c^As per NSW and National PrEP guidelines

^d^Information in relation to eligibility for PrEP

^e^At 3 months and every 6 months thereafter

^f^Consider annual testing for HCV, or more often in men with substantial risk

^g^HIV and STI tests conducted per current standard of care according to the National HIV and STI testing guidelines. Data obtained electronically quarterly from ACCESS study

^h^Behavioural data collected on consenting participants

### Participants

We estimated the number of GBM at high risk of HIV acquisition using data available by the end of 2014. The number of males living in NSW who were aged 16–69 (n_1_ = 2,411,288) was derived from the Australian Bureau of Statistics [29]. According to the nationally representative Second Australian Study of Health and Relationships (ASHR2, 2013), 2.3% of 16 to 69 year-old male respondents in NSW self-identified as gay and 81.9% of these men had been sexually active in the last year [30] (n_2_ = n_1_*0.023*0.819 = 45,422 sexually active gay men in NSW). ASHR2 participants who identified as bisexual were not included in these estimates, because these men were considered much less likely to discuss their homosexual behaviour with clinicians and, ultimately, to be identified as candidates for PrEP assessment. After excluding HIV positive GBM (estimated n_3_ = 6550 [31]), the remaining men (n_4=_ n_2_- n_3=_38,872) represented sexually active men in NSW identifying as gay and at risk of acquiring HIV.

The database of the 2014 Sydney Gay Community Periodic Survey (SGCPS), an annual survey of behaviour among gay community engaged GBM in NSW [32], was used to determine the proportion of GBM at high risk of acquiring HIV who would be eligible for PrEP according to the NSW PrEP guidelines ([24]. Men were considered to be high risk if they reported at least one of the four high-risk behavioural eligibility criteria in the NSW PrEP guidelines. These were related to being a regular partner of an HIV-infected man without HIV viral load suppression, having receptive condomless anal intercourse (CLAI) with casual partners, reporting a recent anal STI diagnosis or syphilis, or methamphetamine use. (Table 1). The SGCPS indicator of having had within the preceding 6 months, ten or more casual partners was used as a proxy indicator for on-going sustained risk within the next 3 months. In total, 8.6% of HIV negative or HIV status unknown 16–69 year-old GBM reported at least one of these criteria. We assumed this proportion to be generalizable to all gay-identifying men in the population, and applied it to the number of sexually active gay-identifying men at risk for HIV in NSW to calculate the minimum number of men eligible for PrEP in NSW (N = n_4_*0.086 = 3343). We recognise that the inclusion in the estimate only of men reporting at least ten casual partners in the previous six-month period is a strict definition of those likely to have on-going risk, and that some men who have had fewer casual partners would also meet the criteria for PrEP eligibility in NSW. Allowing for loss to follow up, the study team considered that enrolling 3700 would represent a sufficiently high proportion of those truly at high risk of HIV infection at the time when PrEP use was not high (recent PrEP use was reported by just 4% of non-HIV-positive men in the 2016 SGCPS [9]). Additionally, it was considered that target enrolment of 3700 participants within existing sexual health services was feasible.

The previously conducted  Australian Health in Men Study found that each of the four high-risk factors were associated with an HIV incidence of above 1.8 per 100 person years [21]. Assuming a similar baseline HIV incidence in the EPIC-NSW cohort, a sample of 3700 participants, with 80% retention over the course of one year, will have 99.2% power to detect a 50% reduction in HIV incidence during the course of the first year on PrEP and 100% power to detect an 84% reduction in HIV incidence (as in European randomised trials of high-risk gay men on PrEP [12, 13]).

### Study medication

All participants receive sufficient supply of daily oral TDF/FTC for self-administration until the next scheduled follow-up visit. A 2000 person-year (PY) supply of TDF/FTC was provided by Gilead Sciences, Inc., and 5400 PY of generic TDF/FTC equivalent were purchased from Mylan Laboratories Ltd. The procurement process was competitive and occurred within a framework for ensuring safety and security of the supply chain, and the quality and bioequivalence of the generic TDF/FTC with Truvada®. Participants’ ongoing access to PrEP after study completion will be facilitated by clinicians at their last study visit.

### Study eligibility

Eligibility for this study is guided by the NSW Health guideline on Pre-Exposure Prophylaxis of HIV with Antiretroviral Medications. To be eligible, participants must be aged 18 years or over, live or visit NSW regularly enough to be able to attend clinics for follow-up assessments, be documented as HIV negative at enrolment or within 7 days of starting PrEP, and be at high and ongoing risk of acquiring HIV through sexual exposure as established by the behavioural risk criteria (see Table 1). Excluded from participation are individuals infected with HIV-1, or with symptoms consistent with acute HIV infection. If HIV status is indeterminate at the enrolment visit, the start of TDF/FTC is delayed for at least 1 month, to confirm HIV negative status. The full list of inclusion and exclusion criteria is presented in Table 3.

**Table 3:** Inclusion and exclusion criteria for participation in the EPIC-NSW study

Inclusion criteria	Exclusion criteria
1. HIV negative at enrolment, with a negative HIV test result documented within 7 days of initiating PrEP	1. HIV-1 infected or has symptoms consistent with acute viral infection (If HIV positive status is not confirmed by testing, delay starting PrEP for at least 1 month and reconfirm negative HIV-1 status).
2. At high and ongoing risk for acquiring HIV infection through sexual exposure (as defined by Behavioural Eligibility criteria presented in Table 1)	2. Having an estimated creatinine clearance (glomerular filtration rate [GFR]) <60 ml/min
3. Aged 18 years or over	3. Having or developing clinical symptoms suggestive of lactic acidosis or pronounced hepatotoxicity (including nausea, vomiting, unusual or unexpected stomach discomfort, and weakness)
4. Live in NSW or visit NSW enough to attend clinics for follow-up assessments	4. Concurrently taking a nephrotoxic agent (e.g., high-dose non-steroidal anti-inflammatory drugs / NSAIDs)
5. Willing and able to provide informed consent	5. Allergic to TDF and/or FTC (based on self-report or recorded)
6. Medicare ineligible individuals may be enrolled if the clinical service is able to cover the costs of monitoring of the patient	6. Concurrently taking prescribed products containing FTC or TDF other drugs containing lamivudine
	7. Factors or conditions that may compromise a participant’s access to health services for follow-up (incarceration or planned relocation and potential absence from NSW for the duration of the study)

### Enrolment of participants

Over 20 clinical sites in urban and regional areas of NSW are participating in the project, including public HIV and sexual health services and private general practices with expertise in HIV antiretroviral medication prescribing (see participating health services in Appendix). Clinical sites are eligible to participate if they: (i) are willing to participate in PrEP roll-out; (ii) can enroll at least 10 patients at high risk for HIV who are eligible for PrEP; (iii) are able and willing to follow the study protocol, and (iv) are participating or willing to participate in the ACCESS study and have a patient management system which enables automated electronic transfer of the study variables, or alternatively are willing to complete manual data entry.

Study promotion materials (e.g., paper and electronic posters, flyers, cards and social media) were developed by NSW’s peak gay and lesbian health organisation, AIDS Council of New South Wales (ACON), in consultation with clinicians, researchers and the NSW Government. Their branding style is consistent with the highly recognisable ‘Ending HIV’ campaign from the NSW Ministry of Health communication framework [33]. Other promotion activities conducted by ACON include establishment and management of a list of people interested in PrEP prior to study initiation, online information about study enrolment, community forums and engagement with specific groups of GBM (e.g., GBM of Asian background). The NSW Sexual Health Infolink (SHIL), a NSW Health service, triages potential participants and conducts referral to participating clinics. The study is also promoted on social media and a variety of websites including the study website (https://epic-nswstudy.org.au), the NSW Health Ending HIV (http://www.health.nsw.gov.au/endinghiv) page, ACON’s website (https://www.acon.org.au/what-we-are-here-for/hiv-prevention) and the websites of some participating clinics. ACON in partnership with PozHet (community organisation of HIV positive heterosexual people) also developed promotional materials on PrEP for the heterosexual community.

Potential participants at high HIV risk are identified among patients attending clinical services based on information available to the clinics, including previously reported high risk behaviour, recent diagnosis of a rectal STI or syphilis, current use of PrEP obtained elsewhere (e.g., purchased online, prescribed in previous demonstration study PrELUDE) or post-exposure prophylaxis (PEP). Some patients are self-referred as a result of promotion or word of mouth, or referred to participating clinical services by other health service providers.

Upon presentation to health services, potential participants receive information about PrEP and the EPIC-NSW from clinicians or trained peer educators from ACON. In some clinics, trained peer educators provide information about PrEP individually or in group sessions. Risk assessment and eligibility screening for PrEP is conducted using a brief online questionnaire by clinicians. An initial assessment may also be done by a peer educator. In all participating sites, trained clinicians (doctors and nurses) conduct initial risk assessment, and two participating sites additionally use trained peer educators from ACON for onsite or outreach recruitment.

Patients willing to proceed with enrolment into EPIC-NSW are asked to provide individual written informed consent (see Additional file 1 for patient information and consent form) and then assessed for all remaining study inclusion and exclusion criteria described in Table 3. The study consent form also includes three separate optional consents to:

(i) participate in a brief quarterly online survey about adherence and risk behaviour;
(ii) link the person’s details to the HIV and STI Registries in NSW, and/or
(iii) be contacted in the future for other research projects.

Participants are able to change or withdraw their consent to participate in any or all aspects of the study at any time during the course of the study.

To ensure that EPIC-NSW provides equitable access to PrEP to all eligible people in NSW, ACON identified the six most commonly used community languages for non-native English speakers in NSW (including Chinese, Thai, Spanish, Indonesian, Portuguese and Arabic). Consent forms and study promotional materials have been translated/back-translated into these six languages.

### Study visits

Study visits and assessment procedures are consistent with the NSW PrEP guidelines (see Table 2). Eligibility screening, enrolment and baseline procedures can be conducted at the same visit. Potential participants provide a medical history (including antiretroviral and other concomitant medications) and undergo testing for HIV (using a 4th-generation HIV test to screen, and supplemental molecular tests if HIV seroconversion is thought possible), STIs (using standard-of-care STI tests, with STI treatment if indicated [34]), hepatitis B and C (using hepatitis B and C antibody tests, with immunisation against hepatitis B if non-immune and not infected), and assessment of renal function. HIV negative status must be confirmed by a fourth generation antibody/antigen HIV test conducted on a specimen collected within 7 days prior to PrEP initiation.

Follow-up visits are conducted three-monthly, in addition to a visit at 1 month after enrolment, to confirm HIV negative status and provide medication adherence advice if necessary. Follow-up visits serve to confirm participants’ ongoing eligibility for PrEP, identify issues with PrEP use, and to screen for HIV and other STIs. Renal function is evaluated at 3 months and biannually thereafter. Hepatitis C antibody testing is conducted annually or more often if clinically indicated. Participants reporting a known or suspected exposure to HIV within 72 h, and a missed daily PrEP dose within 24 h before or after the exposure, are recommended PEP according to the National PEP Guidelines [35].

If eligibility criteria are met and the participant is willing to continue taking PrEP, they receive a TDF/FTC prescription or are dispensed medication for the period until the next study visit. All participants receive supportive adherence counselling and information where necessary.

To support enrolment and follow-up of participants within existing clinic resources, a variety of models of care and study drug provision were supported within EPIC-NSW, including nurse-led models of care, supply and dispensing, and telehealth [36].

### Study endpoints

The primary focus of this study is on evaluating the population-level impact of a rapid, targeted PrEP roll-out on HIV diagnoses among GBM in the EPIC-NSW cohort, and HIV incidence among PrEP study participants. Table 4 presents key endpoints alongside study objectives.

**Table 4:** Study objectives and endpoints

Study objectives	Endpoints
Primary	
i. Assess the incidence of HIV among PrEP study participants	i. Incidence of HIV infection per 100 person-years among study participants
ii. Measure the population-level impact of the rapid roll-out of PrEP on HIV diagnoses among GBM in NSW over a two-year period	ii. The annual number of HIV diagnoses among gay and bisexual men notified to the NSW Ministry of Health in the 12 months following full study enrolment
Secondary	
iii. Evaluate the rate of PrEP uptake among high risk GBM in NSW	iii. Rate of enrolment to the study by clinic type and clinic location
iv. Assess the incidence of STI (gonorrhoea, chlamydia and infectious syphilis) among people prescribed PrEP	iv. Incidence of STI (gonorrhoea, chlamydia and infectious syphilis) per 100 person-years among study participants
v. Describe patterns of PrEP use and medication adherence to the recommended PrEP medication schedule in those prescribed PrEP	v. Patterns of daily TDF/FTC PrEP use and adherence to the medication schedule among study participants (those who opted into brief surveys about adherence and risk behaviour

### HIV Seroconversions and adverse events

To minimise the likelihood of enrolment of individuals with early acute HIV infection, during the screening, clinicians obtain a history of nonspecific signs or symptoms of viral infection during the preceding 30 days and determine the need for a repeat HIV test.

In case of an HIV positive test result during the study, participants will be advised to stop TDF/FTC and managed according to national HIV management guidelines, including genotyping for drug-resistance [37]. Upon the receipt of additional consent, seroconverters will be asked to participate in a qualitative interview to gain detailed information about PrEP adherence and risk behaviours likely to be associated with the transmission event, and to provide biological specimens to allow an assessment of recent TDF/FTC adherence.

EPIC-NSW reports information on serious adverse events, including HIV infections, to the Human Research Ethics Committee (HREC) which approved the study and to the manufacturers of TDF/FTC.

### Data collection

#### Online enrolment database

To mimic ‘real world’ implementation as closely as possible, clinical management information is recorded routinely in clinical records and the study collects only critical information about participants in an online study database, including: date of enrolment, a unique client ID number which is generated automatically by the patient management system, gender, risk assessment information and eligibility for PrEP, and confirmation of informed consent, including consent to each optional study component. This online database is designed in SurveyGizmo (a secure online survey platform, Boulder, Colorado, USA). When the client ID is entered by the clinician, the database automatically converts it to a different number (hereafter referred to as scrambled client ID), a requirement of ethical approval. Only participants who agree to join the optional study components, are asked to provide their full name, date of birth and postcode (for data linkage purposes) and an email address (for online surveys of behaviour and medication adherence).

#### Clinical data collection

All clinical data required to evaluate the uptake and impact of EPIC-NSW is sourced from routine electronic records maintained in the clinical patient management systems. A software program called GRHANITE is used to extract the data. The software was specifically developed to link primary care and hospital data without any personal information leaving the clinic and the tool is used in the ACCESS project [28], to extract and link data from primary care clinics, hospitals and laboratories [38]. Using GHRANITE, data are stripped of any identifying information before leaving the clinical site but can be linked by the scrambled client ID generated by the GHRANITE tool. EPIC-NSW study variables are regularly extracted from clinic databases, and transferred to the ACCESS central database at the Kirby Institute, UNSW Sydney. The variables extracted are: year of birth, country of birth, postcode of residence, Indigenous status, issuing of PrEP prescriptions, STI, HIV, hepatitis and creatinine clearance tests and test results, along with the scrambled client ID and clinic ID. The scrambled client ID is formed by the same conversion process as in the enrolment database. Each quarter, a list of scrambled client IDs from the EPIC-NSW enrolment database are provided to the ACCESS data custodians who retrieve the relevant EPIC-NSW study variables described above. The participating clinics utilising data management systems not compatible with GHRANITE enter the same clinical data in a secure online database developed in SurveyGizmo, and so that information from all clinics is available for monitoring of key indicators.

***Risk assessment and behavioural data collection*** is conducted using separate online survey instruments, also designed in SurveyGizmo. The risk assessment tool is used by clinicians to enter information about the participants’ eligibility for enrolment into EPIC-NSW. As to behavioural data collection, participants who voluntarily opt into this component, receive quarterly email invitations with an automatically generated link to a brief survey (up to 10 min to complete). This survey is focused on a seven–day history of sexual partners and practices, and PrEP dosing. Data collected by this approach enable assessment of the patterns of PrEP use as related to patterns of behaviour and the distinction between non-adherence and intentional intermittent PrEP use. The brief, easy-to-follow format of this survey and two automatic email reminders (sent 1 week apart to non-responders) are designed to maximize the survey participation rates.

### Data analyses

Using data from the EPIC-NSW cohort, we will calculate the incidence of HIV infection per 100 PY after TDF/FTC prescription and the effect-modifying role of non-adherence to the prescribed daily medication schedule. Incident infection is defined as an HIV diagnosis following a negative test, and time at risk calculated as the time between each patient’s first and last test or a patient’s first test and HIV diagnosis. The number of incident infections will be divided by the total person-years at risk. Analyses of HIV seroconversions will also include a period of 12-months after the completion of the study treatment, using data linkage with the NSW HIV registry (for those participants who consent to data linkage). Similarly, we will calculate the incidence per 100 PY for gonorrhoea, chlamydia and syphilis. Comparisons of incident events across groups and over time will be conducted using survival analysis and cox proportional hazards regression methods.

Using routinely available HIV and STI surveillance data in NSW, we will also assess the average quarterly trends of HIV and STI tests and diagnoses in the population, including in the 12-month period immediately prior to the commencement of recruitment (the pre-intervention period), compared to the 12-month period immediately after the recruitment of the sample of 3700 high-HIV risk individuals.

Analyses of sexual practices and adherence to PrEP will be conducted on the subsample of GBM participating in the optional adherence and behavioural survey. Analyses of sexual practices will mainly focus on CLAI. Analyses of adherence to the study medication will concentrate on time on PrEP, proportion and count of days when PrEP pills were taken, temporal changes in CLAI and PrEP use, and the relationship between CLAI and adherence. We will use longitudinal regression methods as appropriate for binary and count data analyses, to compare proportions and counts of events between groups and to assess trends in proportions and event counts over time.

The study Management Team monitors the study enrolment and primary study outcomes against new HIV diagnoses notified to NSW Health and reported each quarter.

Data analysis is conducted by the Kirby Institute research staff using STATA (StataCorp, College Station, TX, USA).

## Discussion

EPIC-NSW is a population-based implementation trial of primary HIV prevention with daily TDF/FTC in the context of sexual HIV exposure. This trial evaluates the population impact of PrEP on the HIV epidemic in the entire state of NSW, Australia. It is distinct from studies which have examined the individual-level preventative effect of TDF/FTC [14].

Internationally, this is one of the first trials which aims to provide PrEP to the entire estimated population of GBM at high risk for HIV infection in a particular jurisdiction. EPIC-NSW follows the NSW PrEP guidelines [24] in identifying individuals at high risk for HIV infection. A targeted high-risk approach can be a viable and cost-effective option in other similar settings with initially limited access to PrEP.

The criteria for PrEP eligibility in EPIC-NSW take into account the local epidemiology of HIV acquisition [4, 39]. Factors with HIV incidence of about 2 per 100 person-years or higher were selected to indicate high risk for HIV acquisition in the Australian context. Indicators from the ongoing behavioral surveillance and other research in local gay communities [30, 32], which measure these criteria, were used to estimate the size of the population of GBM and, specifically, of the group at high risk of HIV which is targeted with PrEP.

The data systems employed in EPIC-NSW allow data to be linked between clinical health services in NSW and with NSW HIV notifications data. The methodology employed allows calculating HIV incidence not only in the EPIC-NSW cohort, but also diagnoses in the underlying population in the state of NSW. This methodology minimizes the data collection burden on health services and is as close to real life as possible. This makes EPIC-NSW one of the first population-based cohorts with an ability to assess the impact of PrEP at the population level.

With a follow-up time of at least 7400 person years, the study presents a unique opportunity to inform the international community about the additive effect of PrEP in HIV prevention.

This trial became possible due to the concerted efforts of the research team at the Kirby Institute, UNSW Sydney, the state government with its time-limited and goal-oriented HIV strategy [10], consensus from clinicians that PrEP implementation was required, a highly functional network of public and private health services, and community activism, leadership and participation in HIV prevention.

### Additional files

Additional file 1: Participant Information Sheet/Consent form. (DOCX 78 kb)

## Appendix

### EPIC-NSW: participating health services

1. Sydney Sexual Health Centre
2. Holdsworth House Medical Practice
3. Royal Prince Alfred Sexual Health Clinic
4. St Vincent’s Hospital NSW
5. Taylor Square Private Clinic
6. Western Sydney Sexual Health Centre
7. Hunter New England Sexual Health Services
8. Clinic 16 (North Shore Sexual Health Clinic)
9. Illawarra Sexual Health
10. Lismore Sexual Health Clinic
  11.East Sydney Doctors
11. Holden Street Clinic, Gosford
12. Short Street Clinic, Kogarah
13. Liverpool Sexual Health Clinic
14. Kirketon Road Centre
15. Nepean Blue Mountains Sexual Health & HIV Clinics
16. Albury Wodonga Health, Albury Sexual Health Service
17. Murrumbidgee Local Health District, Wagga Sexual Health Centre
18. Mid North Coast Local Health District, Area HIV/Sexual Health Services in Coffs Harbour, Port Macquarie and Kempsey
19. Orange Sexual Health Clinic
20. Dubbo Sexual Health Clinic
21. The Albion Centre

## Abbreviations

FTC: Emtricitabine
GBM: Gay and Bisexual Men
HIV: Human Immunodeficiency Virus
HREC: Human Research Ethics Committee
NSW: New South Wales
PrEP: Pre-exposure Prophylaxis
PY: Person-years
STI: Sexually Transmitted Infection
TDF: Tenofovir Disoproxil Fumarate
