# Correction: expanded HIV pre-exposure prophylaxis (PrEP) implementation in communities in new South Wales, Australia (EPIC-NSW): design of an open label, single arm implementation trial

**DOI:** 10.1186/s12889-018-5173-7

**Published:** 2018-02-28

**Authors:** Iryna B. Zablotska, Christine Selvey, Rebecca Guy, Karen Price, Jo Holden, Heather-Marie Schmidt, Anna McNulty, David Smith, Fengyi Jin, Janaki Amin, David A. Cooper, Andrew E. Grulich, on behalf of the EPIC-NSW study group

**Affiliations:** 1 The Kirby Institute, University of New South Wales, Sydney, 2052 Australia; 2 NSW Ministry of Health, Sydney, NSW Australia; 3 AIDS Council of New South Wales (ACON), Sydney, NSW Australia; 4 Sydney Sexual Health Centre, Sydney, NSW Australia; 5 Mid North Coast Local Health District (Area HIV/Sexual Health Services), Lismore Health Service, Lismore, NSW Australia; 6 MacquarieUniversity, Sydney, NSW Australia

## Correction

After publication of the article [1], it has been brought to our attention that one of the members of the EPIC-NSW study group has had their name spelt incorrectly in the acknowledgements. The article mentions “Muhammad Hammoud” when in fact the correct spelling is “Mohamed Hammoud”.
