# Supplementary material for: Expanded HIV pre-exposure prophylaxis (PrEP) implementation in communities in New South Wales, Australia (EPIC-NSW): design of an open label, single arm implementation trial
Source: BMC Public Health. 2018 Feb 2;18:210. doi: 10.1186/s12889-017-5018-9 (PMC5797394; doi:10.1186/s12889-017-5018-9)
Supplement: Supplementary file 1 — Participant Information Sheet/Consent form. (DOCX 78 kb) [file 12889_2017_5018_MOESM1_ESM.docx]

*Site: Insert Header with institution’s name or institution’s letterhead as required*

**Participant Information Sheet/Consent Form**

**Interventional Study** - *Adult providing own consent*

*[Insert site name if required]*

| **Title** | Impact of the rapid expansion of pre-exposure prophylaxis (PrEP) on HIV incidence, in a setting with high HIV testing and antiretroviral treatment coverage, to achieve the virtual elimination of HIV transmission by 2020: a NSW HIV Strategy implementation project |
| --- | --- |
| **Short Title** | EPIC-NSW: Expanded PrEP Implementation in Communities in NSW |
| **Protocol Version** | 2 February 2016, Version 1.2 |
| **Protocol Number** | HEPP 1511 |
| **Project Sponsor** | The Kirby Institute for Infection and Immunity in Society |
| **Coordinating Principal Investigator** | Professor David Cooper, The Kirby Institute |
| **Associate Investigator(s)** | Professor Andrew Grulich, Dr Iryna Zablotska, A/Prof Rebecca Guy, Dr Jeff Jin, A/Prof Janaki Amin, A/Prof Anna McNulty, Dr Mark Bloch, Ms Jo Holden, Dr Christine Selvey, Dr Kerry Chant, Dr Heather-Marie Schmidt, Mr Nic Parkhill. Mr Craig Cooper, Mr Bill Whittaker, Ms Levinia Crooks |

# Part 1: What does my participation involve?

# 1 Introduction

Thank you for taking part in the **EPIC-NSW study** **(Expanded PrEP Implementation in Communities in NSW**). Your participation helps us assess how pre-exposure prophylaxis (PrEP) against HIV helps reduce HIV transmission in NSW. PrEP is not currently widely available in NSW and is being offered here as part of a research study.

You are invited to take part because you are HIV-negative and may be at increased risk of getting HIV (Human Immunodeficiency Virus). If untreated, HIV causes Acquired Immunodeficiency Syndrome, or AIDS. In this study, a medicine that has been used for many years to treat people with HIV is given to HIV-negative people to evaluate its use for lowering their chance of getting HIV. This is called preexposure prophylaxis or ‘PrEP.’ Prophylaxis means doing something to prevent an illness or infection.

This Participant Information Sheet/Consent Form (PICF) tells you about EPIC-NSW. It explains the requirements of this study. It may help you decide if you want to take part in the research.

Please read this information carefully. Ask questions about anything that you don’t understand or want to know more about. Before deciding whether or not to take part, you might want to talk about it with others.

# 2 What is the purpose of this research?

Despite all prevention efforts, many people in Australia continue to be infected with HIV. The NSW government HIV strategy 2015-2020 is aiming to eliminate new HIV cases by the year 2020. This research will evaluate a new additional way to lower people’s chances of getting HIV. It will provide PrEP to people who are at high risk for HIV and evaluate what impact this new prevention approach will have in NSW on the community level.

The drugs being used in EPIC-NSW is called Truvada®(made by the company named Gilead Sciences Inc.). Truvada® is a single tablet made up of two HIV medications: tenofovir disoproxil fumarate and emtricitabine (a combination known as TDF/FTC). Both drugs have been widely used for many years to treat HIV. When used with other medicines in people who already *have* HIV, TDF/FTC reduces the amount of HIV virus in the blood. TDF/FTC does not cure HIV or AIDS, and it is not an HIV vaccine.

As a treatment for people who already have HIV, TDF/FTC is approved for use in most of the world. As a medicine for PrEP, to lower chances of HIV in those who are not infected, TDF/FTC has been approved in the US, but not in Australia. Therefore, in Australia, until TDF/FTC is approved for prevention, this study is considered experimental.

**How well does PrEP work?**

Research in other countries, including the US, has shown that the chances of getting HIV through sex are lowered for people who take PrEP. How well PrEP works depends on how good people are at taking PrEP ***every day***.

There have been 3 main studies around the world which have examined how well PrEP works in lowering the risk of getting HIV in men who have sex with men (iPrEx, IPERGAY and PROUD). They found that the risk of HIV infection was reduced by 44%, 86%, and 86%. There have also been six studies in heterosexual men and women which also showed significant reductions in risk where people were adhering and taking the medicine daily. In the iPrEx study which observed **men who have sex with men for 72 weeks, men who took tablets every day (adherent) and had the PrEP drugs detected in their blood, the drugs were 95% effective.** This means that some people on these research studies still got infected with HIV even though they were taking PrEP. However, only a handful of infections were observed across all studies among people who were taking PrEP every day.

# 3 What does participation in this research involve?

Your involvement in the EPIC-NSW study will last about 24 months (2 years). During this time, you will:

1. Sign a consent form
2. Receive free PrEP medicine for 24 months. Your clinician will advise you to attend your clinic for regular assessments and HIV/STI testing as recommended by NSW PrEP Guidelines.
3. Allow researchers to obtain health information from clinical records (PrEP use, HIV/STI testing and other tests). All your information will be coded, which means that your personal details won’t be included.
4. If you agree, you will receive invitations once every 6 months to complete a brief 15 minute survey. The questions will ask about things like your relationships, sexual practices and other related behaviour and about how often you have taken your study medications since your previous online questionnaire. All your information will be coded, which means that your personal details won’t be included.
5. If you agree, your personal details will be used to check the NSW Registry for any HIV and STI diagnoses which occurred during the study.

The overall study will run for about four years. By entering the study, you are not committed to being in the study and taking PrEP for the whole 24 months. You can stop participating at any time for your own reasons.

**Screening Assessment**

You may have completed a brief internet-based survey, to assess if you are eligible for PrEP. This screening survey did not record any identifiable information but provides a study number which will be used during the study.

The consent form must be signed by you before any study assessments are done.

**Who can take PrEP?**

To start you on PrEP, your clinician will check if you are a good candidate for PrEP. In order to qualify for the study, you must:

- Be at least 18 years old
- Have a negative HIV test within seven days of starting PrEP
- Not show possible signs of having HIV
- Be at high risk for getting HIV
- Be willing and able to consent to this study.

**Who can’t take PrEP?**

You will be advised **NOT** to take PrEP if you

- Have symptoms consistent with acute viral infection
- Have certain kidney or liver problems
- Take certain medications that might make PrEP unsafe
- Have known allergies to TDF/FTC
- You are breastfeeding.

You also cannot be in this study if you have any conditions that would make it difficult for you to take PrEP every day or you cannot attend the clinic for follow-up assessments.

Your clinician will follow the NSW PrEP Guidelines to provide you with due care. They will conduct an HIV test (to confirm that you do not have HIV virus), some tests for gonorrhoea, chlamydia, and syphilis infection (to give you treatment if necessary) and will check to make sure you are not at increased risk of possible side effects from TDF/FTC such as kidney or liver problems. If you are a woman who can get pregnant, you will have a pregnancy test before starting PrEP and while taking it, so that you and your clinician can together make an informed decision whether you start and continue taking PrEP.

Both of the drugs in TDF/FTC work against hepatitis B. If your hepatitis status is not known, you will be tested for hepatitis B to work out the best plan for you. If you have never had hepatitis B, you may be offered a vaccine against it. If you were exposed to hepatitis B previously and the infection has resolved, or you have already been vaccinated, no further action will be necessary. If you have chronic hepatitis B, you will be advised to take TDF/FTC every day to protect you from HIV and keep hepatitis B infection under control. You may also be tested for hepatitis C if this is considered standard at the clinic.

In addition, your clinician may ask you whether you have any symptoms consistent with a new HIV infection and you have experienced any known or suspected exposure to HIV during the last 30 days. If you have, you will receive additional HIV testing, and may be asked to delay starting PrEP until your HIV status is confirmed HIV negative. **It is very important for your safety during this study that you tell the clinician if you are experiencing any of the following symptoms consistent with acute viral infection at screening or before you start PrEP, and also at your follow-up assessments. If you do but it is not time for your regularly-scheduled follow-up assessment, please contact your clinician.**

- Fever
- Feeling tired (fatigue)
- Muscle aches (myalgia)
- Joint aches (arthralgia)
- Skin rash
- Headache
- Sore throat (pharyngitis)
- Night sweats
- Swelling of the lymph nodes around the head and neck (cervical adenopathy)

There may be a few days between the time you start screening for this study, and the time you can start taking PrEP. If you encounter a high-risk sexual event before you start taking TDF/FTC, please tell your clinician immediately, so that your clinician can assess whether you need to receive medication for “Post-Exposure Prophylaxis,” or PEP before you start taking PrEP. This will not affect your ability to start taking PrEP once you finish with PEP if you are eligible.

**Starting PrEP**

After you have met all study criteria, you will be given a prescription for 30 days of TDF/FTC and asked to start taking it as PrEP immediately. However, if your HIV test result is not available yet, you will start taking it after your clinic will notify you that your test result is negative and you are okay to start it. Your clinician will provide you with information to help you remember to take it every day. You will also receive information about safer sex practices including condoms.

It is important to know that your clinician will recommend:

- You take PrEP every day, as per the instructions
- You practice safer sex
- You attend the clinic for regular HIV/STI testing and other assessments as recommended by the NSW PrEP Guidelines.

**How do I take PrEP?**

The current clinical guidelines recommend you take one PrEP tablet every day. If there are any changes to this recommendation your clinician will let you know. A tablet of TDF/FTC must be taken by mouth, at about the same time every day, and it is recommended to be taken with food. You may continue to take PrEP every day for up to 24 months in total.

If you miss a dose of TDF/FTC, take the missed pill as soon as you remember on that same day, but do not take more than two doses of TDF/FTC in a 24 hour period. Contact your clinician as soon as possible if you take more than two pills of TDF/FTC a day, to avoid overdosing.

TDF/FTC should be stored at room temperature in its original container. The container should be kept tightly closed and out of the reach of children. Do not give TDF/FTC prescribed to you to other people.

**Follow-up clinical assessments while taking PrEP**

Your clinician will advise you of the tests and assessments recommended for people taking PrEP in accordance with guidelines. These assessments are not a requirement of participating in the study but are important for your health. These assessments include tests for HIV (every three months with an additional test at one month after starting PrEP), sexually transmitted infections (every three months), and sometimes for your kidney health (three months after you start PrEP and every six months after that). If you are a woman who can get pregnant, you will also be advised to take a pregnancy test every three months. You may also be given other services, or referral for any services, if needed for your health.

If you are willing to continue taking PrEP you will be given a new prescription for TDF/FTC at the next assessment. All of these follow-up procedures are considered standard of care for people who are at risk for HIV infection and/or are taking anti-HIV medications as PrEP. This means that even if PrEP wasn’t considered research, these things would be done for your health and safety.

**Stopping PrEP**

It is your right to decide to stop taking PrEP at any time. Your clinician may also have you stop the medication if s/he feels it is not safe for you. This may happen if you become HIV positive, start to experience kidney, liver or other unacceptable health problems, or need medication that is not safe to be taken alongside with TDF/FTC.

Your clinician will discuss with you how to plan to stop PrEP during the study period if you need to. If you become HIV positive during this study, the results of any additional tests about your HIV infection, such as tests of resistance to different HIV treatments, will be collected for this study.

**Additional costs and reimbursement**

You will not be paid and there will be no additional costs to you to be in this project. You will not be reimbursed for your travel to the clinics, clinic parking, your time, or other expenses associated with the research project. While in the study, you will receive the PrEP medication free-of-charge up to 24 months. All tests and medical care will be provided to you as per routine health service arrangements which may be through Medicare, health insurance or other arrangements.

# 4 What do I have to do?

Participation in this study does not require any changes to your diet or participation in sports. It is important to let the clinician know any medications you are already taking or start taking while on TDF/FTC.

# 5 Other relevant information about the research project

Participants in this study will be adults who are HIV-negative and are at risk of getting HIV through sex. The study allows up to 7400 person-years of PrEP (that is 3700 participants for 24 months or two years each).

The EPIC-NSW study is open to all adults regardless of sex, gender or sexual preference who meet the inclusion criteria. There will be about 25 sites involved in the project.

The EPIC-NSW Study Management Team is responsible for the day-to-day management and coordination of the study. Each clinic in the study also has investigators who are part of the study.

# 6 Do I have to take part in this research project?

You do not have to be in this project if you do not want to. If you decide to be in this project now, it is your right to change your mind later. You are free to withdraw at any time. If you do decide to take part, you will be given this Participant Information and Consent Form to sign and you will be given a signed copy to keep.

Your decision whether to take or not to take part in this study, or whether to withdraw, will not affect your routine treatment, your relationship with those treating you, or your relationship with [*insert Institution*].

# 7 What are the alternatives to participation?

You do not have to be in this research project to receive treatment at this clinic. PrEP can be prescribed outside of this project. Other options to prevent HIV include: advice and counselling on safe sex practices including the use of condoms, and HIV post-exposure prophylaxis (PEP) is also available at your clinic. Your clinicians will discuss these options with you before you decide whether or not to take part in this research project.

Other anti-HIV medications are currently being studied for use as PrEP. Follow your clinician’s recommendations as to how to use PrEP.

# 8 What are the possible benefits of taking part?

There are substantial benefits from this implementation research to both the participants and the larger community in NSW. You and every participant will receive access and can benefit from a medicine that is already known from previous research to significantly reduce the risk of HIV infection, but is not available for sale in Australia. Some participants may experience direct health benefits due to testing for HIV and STIs more regularly than they would normally otherwise. By taking PrEP and testing regularly, you will not only benefit yourselves, but will also protect you partners from HIV and pass benefits to others by breaking chains of HIV transmission – a substantial and very important gift to the health of friends and communities. Participants may also like the idea that they are contributing to new knowledge about the impact this new HIV prevention approach may have on HIV epidemic in NSW.

# 9 What are the possible risks and disadvantages of taking part?

Medications often cause side effects. You may have none, some, or all of the effects known to be associated with the use of TDF/FTC and listed below. They may be mild, moderate or severe. If you have any of these side effects, or are worried about them, talk with your clinician. Your clinician will also be looking out for side effects. Many side effects go away shortly after treatment ends. However, sometimes side effects can be serious, long lasting or permanent. If a severe side effect or reaction occurs, your clinician may need to stop your treatment. Your clinician will discuss the best way of managing any side effects with you.

**Possible Side Effects of** TDF/FTC

Possible side effects of TDF/FTC are described in the TDF/FTC product information attached to this form. Please make sure you read it carefully and ask your clinician any questions you may have.

I**n people who did not have HIV and took TDF/FTC for PrEP:**  In previous research studies, between 3% and 9% (or between 3 and 9 in 100) of such participants reported the following four side effects, and these levels were higher than among the participants who did not take TDF/FTC (took a “sugar pill”):

- Headache
- stomach pain
- involuntary weight loss
- nausea or upset stomach

In PrEP studies where these side effects were reported, these were modest, usually only occurred in the first one or two months of taking the drug and did not last.

**Possible allergic reaction:** In clinical trials, a small number of people have had an allergic reaction to tenofovir, one of the drugs in TDF/FTC. Altogether, only eight cases have been reported. Symptoms of an allergic reaction may include fever, rash, upset stomach, vomiting, loose or watery stools, stomach pain, achiness, shortness of breath, a general feeling of illness or a potentially serious swelling of the face, lips, and/or tongue.

The company that makes TDF/FTC also warns that the following changes in laboratory tests are possible. This information is based mostly on experience with people who take TDF/FTC for HIV treatment, and on other drugs that are like TDF/FTC. Because HIV by itself can also cause these same problems, it is difficult to know how much TDF/FTC alone contributes to kidney and bone disease:

- kidney problems
- decreases in the minerals in their bones
- build-up of lactic acid in the blood
- enlarged liver

**Call your clinician immediately if you get these symptoms:**

| **Symptoms of too much lactic acid in the blood** | **Symptoms of severe liver problems** |
| --- | --- |
| • weakness or being more tired than usual  • unusual muscle pain  • being short of breath or fast breathing  • nausea, vomiting, and stomach-area pain  • cold or blue hands and feet  • feel dizzy or lightheaded  • fast or abnormal heartbeats | - - your skin or the white part of your eyes turns yellow   - dark “tea-coloured” urine   - light-coloured stools   - loss of appetite for several days or longer   - nausea   - stomach-area pain |

**To manage kidney and liver health:** Your clinician will be taking blood tests to check you kidneys and liver before you start and while you are taking TDF/FTC. Your clinician may tell you to stop taking TDF/FTC if you develop kidney or liver problems during the study.

**Bone health:**

Only two previous PrEP research studies in HIV negative people on TDF/FTC conducted bone scans (one used TDF/FTC and one used TDF alone). Only 1 in 100 study participants tested experienced a small decrease in the minerals in their bones during the first few months of PrEP. This decline either stopped getting worse or returned to normal after the first few months. No increase in bone breaks was observed. According to the NSW Interim PrEP guidelines, bone scans are not recommended for HIV negative people in this study. However, if you have a history of bone fractures/breaks or if you are at significant risk for osteoporosis, please tell the clinician.

**HIV infection**

People with HIV need full HIV medication and treatment. You will have an HIV test at every follow-up assessment. If you become infected with HIV, your clinician will have you stop taking PrEP immediately. This is because taking TDF/FTC by itself when you have HIV can make the virus resistant to some HIV drugs so that they may no longer work for you and limit your HIV treatment options over time.

**Hepatitis B infection**

If you become infected with Hepatitis B while you are taking TDF/FTC, please tell your clinician immediately, and do not stop taking TDF/FTC on your own. If you stop TDF/FTC while you have hepatitis B, your hepatitis symptoms may get worse.

**Pregnancy and breastfeeding**

The effects of TDF/FTC on an unborn child or a newborn are not well known. You may not participate in this study if you are breast-feeding. If you can possibly get pregnant, you must also take a pregnancy test as your clinician recommends. All participants are expected to continue using safer sex practices [non-Catholic sites may add: including the use of condoms].

Any participants who are taking PrEP in conjunction with conception must take daily doses of TDF/FTC beginning one month before a conception attempt and continue until one month after the last attempt to conceive. If you become pregnant during this study, you should tell your clinician immediately. Your clinician will discuss with you your risks of HIV infection during pregnancy, the possible risks of TDF/FTC on your pregnancy and child, and whether or not you should stay in the study. If you become pregnant, we may ask for copies of or access to the parts of your medical records that discuss your pregnancy, delivery, and your infant’s health.

**Other risks**

Although your information will be kept confidential by your clinic and the researchers, it is possible that your friends, family or people in your community may find out that you are in this study, and you may experience stigma as a result of being involved in a study about HIV. In the event you become upset or distressed your clinician will be able to arrange for counselling or other appropriate support which will be provided by appropriately qualified staff.

# 10 What will happen to my samples?

No samples are collected by this study. All study assessments, including testing are conducted according to the NSW PrEP Guidelines.

# 11 What if new information arises during this research project?

If during this project, new information becomes available about the study medication or its use, your clinician will tell you about it and will discuss with you whether you want to continue, and you may be asked to sign an updated consent form that has the new information in it.

# 12 Can I have other treatments during this research project?

It is not anticipated that PrEP will affect any other medications you may be taking. However, it is important to let a clinician know about any other medications or treatments you may be taking and/or using. Your clinician and the TDF/FTC product Information will explain to you which treatments or medications cannot be used concurrently with TDF/FTC.

# 13 What if I withdraw from this research project?

If you decide to withdraw from this project (meaning that you do not want to take PrEP any longer and do not want your data to be collected), please let a member of the research team know before you stop participating. This will allow you to find out about any possible health risks or special requirements before you stop. You should be aware that data collected up to the time you withdraw will form part of the research project results. If you do not want this to happen, you must make a note of this on your consent form.

# 14 Could this research project be stopped unexpectedly? What happens when the study ends?

This research project may be stopped unexpectedly for reasons which may include:

- Unacceptable side effects
- Decisions by local regulatory/health authorities.

PrEP will be available to you for up to 24 months. At the present time TDF/FTC is not registered for use in Australia. At the end of the study, if the study drug is still not registered in Australia AND subsidised on the PBS for your condition, then [*insert study site name*] will not be able to fund ongoing supplies of the drug.

We estimate that all participants in the study will have completed the study by the end of 2018. After this time, the data will be analysed and a summary of results will be posted on the study website.

# Part 2: How is the research project being conducted?

# 15 What will happen to information about me?

**How information is used and stored**

Any information collected about you during this study will remain confidential.

Information collected for this research will be kept in two different databases:

***Health information:*** Researchers will obtain your health information from clinical records at this and other clinics/hospitals or laboratories for the purpose of this research. *The health information will include PrEP prescription, HIV and STI tests and kidney tests recommended in the guidelines. This information will be extracted from the clinical database via the ACCESS study (see ACCESS information sheet) using software which enables it to be provided to the researchers in a coded form. No personal details will be included*.

***HIV and STI diagnoses:*** At the end of the study, researchers will use your personal details to check the NSW Registry to see if any new HIV and STI diagnoses occurred during the study. This is important to assess how well PrEP is working and also if STI infections are increasing because of changes in sexual risk behaviour. Once the check is complete, your personal details will be removed.

***Behavioural survey information:*** Only the researchers at the Kirby Institute will have access to your internet-based survey information which will be stored in a secure password-protected database (SurveyGizmo). The behavioural survey system will store your preferred email address so that the system can send you invitations and automatic reminders to compete the surveys. Because your email address is stored only for follow-up purposes, it will be erased from the system as soon as you stop or complete your study participation.

All electronic databases kept at the Kirby Institute will be protected by passwords and University of New South Wales firewalls. All participants will be allocated a study number, and all electronic data will be stored under this code. Your name and identifying information will not be stored with any other information collected about you during the study.

Your information will only be used for the purpose of this research, and it will only be disclosed with your permission, except as required by law. Coded data may be used in future related research.

**Inspection of research records**

It is possible that your health research study records may be inspected in order to make sure that the study is being conducted appropriately and safely, and that the information is being reported truthfully. Possible inspectors include: representatives from the Kirby Institute, relevant authorities and authorised representatives of the Kirby Institute, the institution relevant to this Participant Information Sheet, *[Name of institution],* or as required by law. By signing the Consent Form, you authorise release of, or access to, this confidential information to these relevant study personnel and regulatory authorities.

**Publication and presentation of research findings**

The findings from this research project will be published and/or presented in a variety of places, most likely combined with everyone else’s information. In any publication and/or presentation, information will be provided in such a way that you cannot be identified.

**Your rights to your information**

In accordance with relevant Australian and New South Wales privacy and other relevant laws, you have the right to request access to your information collected and stored by the research team. You also have the right to request that any information with which you disagree, be corrected. Please contact the study team member named at the end of this document if you would like to access your information.

**Information storage after the study**

Your study files will be archived in a locked storage facility and kept for at least 15 years after the end of the study. Coded data in electronic form will also be stored for this amount of time.

# 16 Complaints and compensation

If you suffer any injuries or have complications as a result of this research project, you should contact the study team as soon as possible and you will be assisted with arranging appropriate medical treatment.

You may have a right to take legal action to obtain compensation for any injuries or complications resulting from your participation in the study.  Compensation from the sponsor of this study, the University of New South Wales, may be available if your injury or complication is caused by the drugs or procedures, or by the negligence of any of the parties conducting the study. If you receive compensation that includes an amount for medical expenses, you will be required to pay for your medical treatment from those compensation monies.

If you are not eligible for compensation for your injury or complication under the law, but are eligible for Medicare, you can receive any medical treatment required to treat the injury or complication, free of charge, as a public patient in any Australian public hospital.

The people running this study agree to follow the Medicines Australia *Guidelines for Compensation for Injury Resulting from Participation in an Industry-Sponsored Clinical Trial*. These Guidelines allow for some claims for compensation to be settled without the need for legal action to be taken. The fact that the people running this study have agreed to abide by these guidelines in respect of the clinical trial does not affect your rights to pursue legal action in respect of any injury you may suffer as a result of participation.  You can obtain a copy of these Guidelines from the Secretary of the Human Research Ethics Committee.”

# 17 Who is organising and funding the research?

This research project is being conducted by the Chief Investigator, Professor David Cooper at the Kirby Institute, and is being funded by the NSW Ministry of Health. The manufacturer of Truvada®, Gilead Sciences Inc. (or simply Gilead), is providing the study drug.

[*Insert site name*] will receive payments from the NSW Ministry of Health for time spent undertaking this research project. No member of the research team will receive personal financial benefit from your involvement in this research project (other than their ordinary wages).

Gilead may benefit financially from this research project if, for example, the project assists Gilead to obtain approval for Truvada® to be used as PrEP in Australia.

In addition, if knowledge acquired through this research leads to discoveries that are of commercial value to Gilead , the clinicians or their institutions, there will be no financial benefit to you or your family from these discoveries.

# 18 Who has reviewed the research project?

All research in Australia involving humans is reviewed by an independent group of people called a Human Research Ethics Committee (HREC). The ethical aspects of this research project have been approved by the HREC of the St. Vincent’s Hospital, Darlinghurst NSW. This project will be carried out according to the National Statement on Ethical Conduct in Human Research (2007, last updated 14 May 2015). This statement has been developed to protect the interests of people who agree to participate in human research studies.

# 19 Further information and who to contact

The person you may need to contact will depend on the nature of your query. If you want any further information about this project or if you have any medical problems which may be related to your involvement in the project (for example, any side effects), you can contact the study chief investigator Professor David Cooper on 9385-0900 or any of the following people:

**Clinical contact person (for each study site to complete; may provide more than one if necessary)**

| Name | *[Name]* |
| --- | --- |
| Position | *[Position]* |
| Telephone | *[Phone number]* |
| Email | *[Email address]* |

For matters relating to research at the site at which you are participating, the details of the local site complaints person are:

**Complaints contact person (for each study site to complete)**

| Name | *[Name]* |
| --- | --- |
| Position | *[Position]* |
| Telephone | *[Phone number]* |
| Email | *[Email address]* |

If you have any complaints about any aspect of the project, the way it is being conducted or any questions about being a research participant in general, then you may contact:

| Reviewing HREC name | St. Vincent’s Hospital Sydney HREC |
| --- | --- |
| HREC Reference number | HREC/15/SVH/436 |
| HREC Executive Officer | Contact person - HREC Executive Officer |
| Telephone | 8382 2075 |
| Email | research@stvincents.com.au |

**Reviewing HREC approving this research** **and HREC Executive Officer details**

**Local HREC Office contact (Single Site -Research Governance Officer – for each study site to complete)**

| Name | *[Name]* |
| --- | --- |
| Position | *[Position]* |
| Telephone | *[Phone number]* |
| Email | *[Email address]* |

**Consent Form**

| **Title** | Impact of the rapid expansion of pre-exposure prophylaxis (PrEP) on HIV incidence, in a setting with high testing and antiretroviral treatment coverage, to achieve the virtual elimination of HIV transmission by 2020: A NSW HIV Strategy demonstration project. |
| --- | --- |
| **Short Title** | The EPIC-NSW Study: Expanded PrEP Implementation in Communities in NSW |
| **Protocol Version** | 2 February 2016, Version 1.2 |
| **Protocol Number** | HEPP 1511 |
| **Project Sponsor** | The Kirby Institute for Infection and Immunity in Society |
| **Coordinating Principal Investigator** | Professor David Cooper, The Kirby Institute |
| **Associate Investigator(s)** | Professor Andrew Grulich, Dr Iryna Zablotska, A/Prof Rebecca Guy, Dr Jeff Jin, A/Prof Janaki Amin, A/Prof Anna McNulty, Dr Mark Bloch, Ms Jo Holden, Dr Christine Selvey, Dr Kerry Chant, Dr Heather-Marie Schmidt, Mr Nic Parkhill. Mr Craig Cooper, Mr Bill Whittaker, Ms Levinia Crooks |

**Declaration by Participant: Consent to be in the EPIC-NSW Study**

I have read the Participant Information Sheet or someone has read it to me in a language that I fully understand.

I understand the purposes, procedures and risks of the research described in the project.

I have had an opportunity to ask questions and I am satisfied with the answers I have received.

I freely agree to participate in this research project as described and understand that I am free to withdraw at any time during the study without affecting my future health care.

I give permission for the researchers to access my health information (PrEP prescription, HIV and STI tests and other tests recommended in the guidelines) from this clinic, other clinics, hospitals and laboratories for the purposes of this project. I understand that such information will not include personal details and will remain confidential.

I understand that I will be given a signed copy of this document to keep.

I understand that if I decide to discontinue the study treatment, the investigator/sponsor will be able to access my health information for collection of follow-up information for research and analysis.

Please ***initial*** one option for the following (yes or no):

I give permission to be contacted by email with an invitation to participate in online surveys of behaviour.

(initial one option) __________Yes __________No

I give permission to link my details with Commonwealth and State agency health and disease-related registries with the approval of state-based Population and Health Services Research Ethics Committee. Identifiers may include my full name, date of birth and postcode of residence. Such registries include, but are not limited to, registries of HIV diagnoses and sexually transmissible infections (STI). This information will allow the calculation of rates of HIV and STI diagnoses in study.

(initial one option) __________Yes __________No

I give permission to be contacted by the Kirby Institute in the future to provide information on changes in my health and lifestyle and be invited to participate in future studies of relevance to this study.

(initial one option) __________Yes __________No

|  | | | | | | |
| --- | --- | --- | --- | --- | --- | --- |
|  | Name of Participant (please print) | |  |  |  |  |
|  | | | | | | |
|  | Signature |  | | Date |  |  |
|  | | | | | | |

|  | | | | | | |
| --- | --- | --- | --- | --- | --- | --- |
|  | Name of Witness* to Participant’s Signature (please print) | |  | | |  |
|  | | | | | | |
|  | Signature |  | | Date |  |  |
|  | | | | | | |

* Witness is not to be the investigator, a member of the study team or their delegate. In the event that an interpreter is used, the interpreter may not act as a witness to the consent process. Witness must be 18 years or older.

**Declaration by Investigator /Senior Researcher**

I have given a verbal explanation of the research project, its procedures and risks and I believe that the participant has understood that explanation.

|  | | | | | | |
| --- | --- | --- | --- | --- | --- | --- |
|  | Name of Investigator /  Senior Researcher^†^ (please print) | |  | | |  |
|  | | | | | |  |
|  | Signature |  | | Date |  |  |
|  | | | | | | |

^†^ A senior member of the research team must provide the explanation of, and information concerning, the research project.

Note: All parties signing the consent section must date their own signature.

**Form for Withdrawal of Participation**

| **Title** | Impact of the rapid expansion of pre-exposure prophylaxis (PrEP) on HIV incidence, in a setting with high testing and antiretroviral treatment coverage, to achieve the virtual elimination of HIV transmission by 2020: A NSW HIV Strategy demonstration project |
| --- | --- |
| **Short Title** | The EPIC-NSW Study: Expanded PrEP Implementation in Communities in NSW |
| **Protocol Version** | 2 February, 2016, Version 1.2 |
| **Protocol Number** | HEPP 1511 |
| **Project Sponsor** | The Kirby Institute for Infection and Immunity in Society |
| **Coordinating Principal Investigator** | Professor David Cooper, The Kirby Institute |
| **Associate Investigator(s)** | Professor Andrew Grulich, Dr Iryna Zablotska, A/Prof Rebecca Guy, Dr Jeff Jin, A/Prof Janaki Amin, A/Prof Anna McNulty, Dr Mark Bloch, Ms Jo Holden, Dr Christine Selvey, Dr Kerry Chant, Dr Heather-Marie Schmidt, Mr Nic Parkhill. Mr Craig Cooper, Mr Bill Whittaker, Ms Levinia Crooks |

**Declaration by Participant**

I wish to withdraw from participation in the above research project and understand that such withdrawal will not affect my routine treatment, my relationship with those treating me or my relationship with *[insert clinic/Institution]*.

|  | | | | | | |
| --- | --- | --- | --- | --- | --- | --- |
|  | Name of Participant (please print) | |  |  |  |  |
|  | | | | | | |
|  | Signature |  | | Date |  |  |
|  | | | | | | |

*In the event that the participant’s decision to withdraw is communicated verbally, the Clinician/Senior Researcher will need to provide a description of the circumstances in the participant’s source documentation.*

**Declaration by Investigator /Senior Researcher^†^**

I have given a verbal explanation of the implications of withdrawal from the research project and I believe that the participant has understood that explanation.

|  | | | | | | |
| --- | --- | --- | --- | --- | --- | --- |
|  | Name of Investigator/  Senior Researcher^†^ (please print) | |  | | |  |
|  | | | | | |  |
|  | Signature |  | | Date |  |  |
|  | | | | | | |

^†^ A senior member of the research team must provide the explanation of and information concerning withdrawal from the research project.

Note: All parties signing the consent section must date their own signature.
